# Oncogene SCARNA12 as a potential diagnostic biomarker for colorectal cancer

**DOI:** 10.1186/s43556-023-00147-x

**Published:** 2023-11-01

**Authors:** Hong Zhang, Xin Liu, Wencheng Zhang, Jiarong Deng, Chuxian Lin, Zhenhua Qi, Yaqiong Li, Yongqing Gu, Qi Wang, Liping Shen, Zhidong Wang

**Affiliations:** 1 Graduate Collaborative Training Base of Academy of Military Sciences, Hengyang Medical School, University of South China, Hengyang, 421001 Hunan China; 2 Department of Radiobiology, Beijing Key Laboratory for Radiobiology, Beijing Institute of Radiation Medicine, Beijing, 100039 China

**Keywords:** CRC, SCARNA12, Oncogene, PHB2, PI3K/AKT pathway

## Abstract

Colorectal cancer (CRC) is one of the most common malignant tumors of the digestive system, and represents a severe threat to the life and health of individuals. Increasing evidence supports the role of small nucleolar RNAs (snoRNAs) as critical regulatory gene in cancer development. Small Cajal body-specific RNAs (scaRNAs), a subtype of snoRNAs, are named for their subcellular localization within Cajal bodies. SCARNA12, which located at the intronic region of PHB2 in chromosome 12p13.31 with 270 nucleotides (nt) in length. It has been reported function as a diagnostic marker for cervical cancer. However, its biological functions and molecular mechanisms in CRC have yet to be elucidated. In this study, bioinformatics analysis revealed that SCARNA12 was highly expressed in CRC and positively correlated with poor prognosis in CRC patients. Additionally, SCARNA12 showed upregulated expression in CRC cell lines and clinical CRC tissue samples. Moreover, SCARNA12 overexpression in SW620 cells accelerated cell proliferation, suppressed the apoptosis rate, and enhanced tumorigenesis in vivo. The knockdown of SCARNA12 expression in HCT116 and HT29 cells resulted in contrasting effects. The functioning of SCARNA12 is mechanically independent of its host gene PHB2. Notably, the overexpression of SCARNA12 activated PI3K/AKT pathway in SW620 cells, and the malignancy degree of CRC cells was attenuated after treatment with MK2206 (a specific AKT inhibitor). Our findings demonstrated that SCARNA12 plays an oncogenic role in CRC progression and can be used as a potential diagnostic biomarker for CRC.

**Supplementary Information:**

The online version contains supplementary material available at 10.1186/s43556-023-00147-x.

## Introduction

Colorectal cancer (CRC) refers to a malignancy of the digestive system. It ranks third among cancers in the global cancer statistics and is the second leading cause of cancer-related death [1, 2]. In 2021, approximately 149,500 individuals in the United States were diagnosed with CRC and 54,410 died of this disease [1]. Patient outcomes can be improved with early detection and regular screening via stool testing or structural exams. Data show that 20% of patients diagnosed with CRC exhibit metastasis [3]. The 5-year survival rate is estimated to be as high as 91% for patients with early-stage CRC, compared to only 15% for patients with metastatic CRC [4]. At present, a combination of surgery with chemotherapy and/or radiotherapy is the most common treatment for CRC. Despite the improvements in CRC diagnosis (e.g., molecular biomarkers) and therapies (e.g., immunotherapy and targeted therapy), many CRC patients receive an unsatisfactory prognosis as the disease was detected at a late stage, and cannot receive personalized treatment. Hence, it is necessary to identify novel biomarkers for the clinical evaluation and predictive analysis of CRC patients.

The carcinogenesis of CRC starts from the appearance of abnormal crypts, gradually evolves into carcinoma precursor lesions (a polyp) and eventually progresses to CRC over a period of 10–15 years. Overall, 70%-90% of CRCs originate from colorectal adenoma, whereas 10%-20% develop from serrated neoplasia [5]. Although the mechanism of CRC carcinogenesis has been studied for a long time, it still remains unclear. To the best of our knowledge, the mutations and/or dysregulation of oncogenes (e.g., KRAS, BRAF, and PIK3CA) [6] or antioncogenes (e.g., APC and PETN) [7] play an important role in the pathological process of CRC by activating carcinogenic signaling pathways, among which the PI3K/AKT pathway is an important one [8]. Aberrant activation of the PI3K/AKT pathway is a major feature in the process of driving CRC carcinogenesis [9]. The PI3K/AKT pathway participates in multiple biological processes including cell proliferation, survival, metastasis, and metabolism [10]. The targeting of the PI3K/AKT pathway is regarded as a viable method for treating CRC and other cancers [11].

Small nucleolar RNAs (snoRNAs) are non-coding RNAs generally distributed in the nucleolus. Small Cajal body-specific RNAs (scaRNAs) are a subtype of snoRNAs first identified in 1984 [12]. They share conserved structural domains, and H/ACA and C/D boxes, and have similar biological functions [13, 14]. The typical role of snoRNAs (including scaRNAs) is to guide pseudouridylation and 2′-O-methylation of targeted RNAs (mainly small nuclear RNAs and ribosomal RNAs) through H/ACA box and C/D box [15–17]. Despite the similarities, some differences exist. SnoRNAs generally located in the nucleolus contain only one type of box, that is, either H/ACA box or C/D box, while scaRNAs usually include two H/ACA box or C/D box or one of each [18]. Additionally, scaRNAs also contain a Cajal body box (CAB box) that directs them to the specific Cajal body, where scaRNAs are assembled and perform their function [19, 20]. In the past two decades, an increasing number of studies have revealed that snoRNAs regulate carcinogenesis at multiple levels and have attractive prospects in the clinical management of cancer, which has become an area of interest in tumor biology research. Till date, several snoRNAs are dysregulated in CRC and function as oncogenes or cancer-suppressor genes (e.g., SNORD12C [21], SNORA21 [22], SNORA24 [23], SNORA42 [24], SNORD44 [25], SNORD57 [26], and SNORD78 [21]). Additionally, researchers have found that the dysregulation of scaRNAs was involved in the progression of a few cancers, including CRC [27–30]. Thus, they could act as candidate biomarkers or therapeutic targets in cancer. However, only a limited number of studies have focused on the function of scaRNAs in carcinogenesis.

Small Cajal body-specific RNA12 (SCARNA12) is processed from the intron of its host gene prohibitin2 (PHB2). It contains a H/ACA box and a C/D box and guides the pseudouridylation of residue U46 in the U5 small nuclear RNA. Previous studies have shown that SCARNA12, in combination with other non-coding RNAs, could serve as a biomarker for cervical cancer screening [31]. Nevertheless, the function and clinical value of SCARNA12 in CRC are still unclear. The expression levels and biological effects of SCARNA12 in CRC were analyzed in this study. Our data indicated that SCARNA12 expression was upregulated in CRC; meanwhile, there was a notable negative correlation between the SCARNA12 level and the prognosis of CRC patients. It was found that SCARNA12 regulates CRC cell proliferation, survival, and xenograft growth by activating the PI3K/AKT pathway. Briefly, our findings provide insight into how SCARNA12, a novel oncogene, may serve as a diagnostic biomarker in CRC.

## Results

### SCARNA12 was highly expressed in CRC

**Fig. 1:**
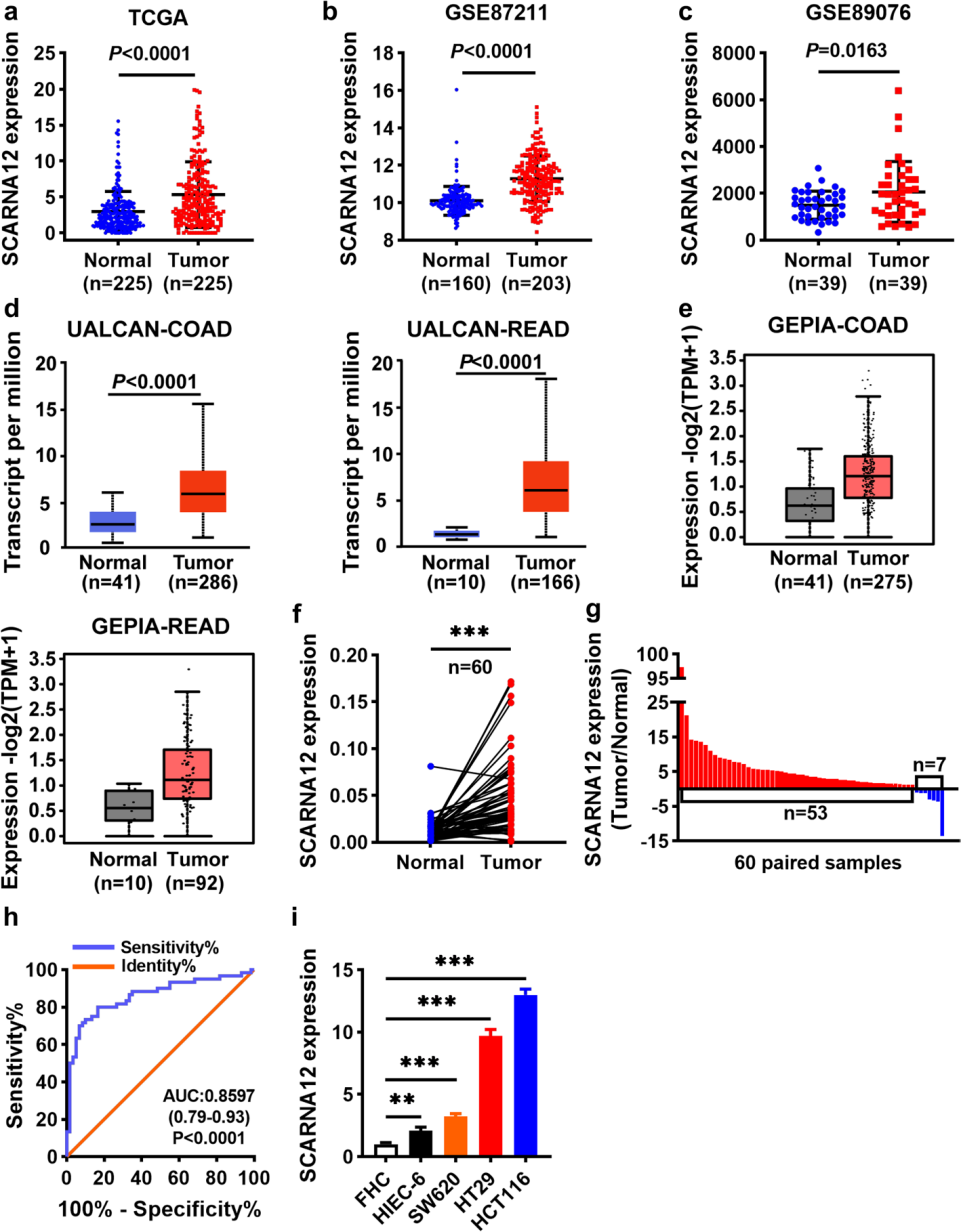
High SCARNA12 expression in CRC tissues and CRC cell lines. **a-e** Relative expression of SCARNA12 in CRC tissues and normal tissues from TCGA database (*n* = 225, each group) (**a**)**,** GEO GSE87211 dataset (*N* = 160; T = 203) (**b**)**,** GEO GSE89076 dataset (*n* = 39, each group) (**c**)**,** UALCAN database (COAD: *N* = 41, T = 286; READ: *N* = 10, T = 166) (**d**)**,** GEPIA database (COAD: *N* = 41, T = 275; READ: *N* = 10, T = 92) (**e**). **f-g** The expression levels of SCARNA12 in 60 pairs of CRC tissues and matched para-cancer tissues were detected by qRT-PCR (**f**). Red columns indicate upregulated and the blue represent downregulated in **g**. **h** The ROC curve of SCARNA12 for distinguishing patients with CRC and normal controls (*n* = 60, each group). **i** QRT-PCR was used to detect the relative expression of SCARNA12 in FHC, HIEC-6, SW620, HT29, HCT116. The results are presented as the mean ± SD (*n* = 3). N, normal; T, tumor; ** *P* < 0.01; *** *P* < 0.001

To explore the role of SCARNA12 in CRC, we analyzed the expression level of SCARNA12 in CRC and adjacent normal tissue samples from the cancer genome atlas (TCGA) database (*P* < 0.001, Fig. 1a), gene expression omnibus (GEO) database, GSE87211 (*P* < 0.001, Fig. 1b), GSE89076 (*P* = 0.0163, Fig. 1c), UALCAN database (*P* < 0.001, Fig. 1d) and gene expression profiling interactive analysis (GEPIA) database (Fig. 1e). The results indicated that SCARNA12 was notably upregulated in CRC tissue samples, as compared to adjacent normal tissues. Furthermore, the GEPIA database showed that SCARNA12 was highly expressed in various tumors (Fig. S1). To confirm the above results further, qRT-PCR was performed to analyze the expression level of SCARNA12 in 60 pairs of clinical CRC samples. The results showed that the SCARNA12 expression level was notably increased in 88% (53/60) of CRC tumor tissues (Fig. 1f, g). Furthermore, CRC tissues could be distinguished from normal mucosal tissues based on the expression level of SCARNA12 via receiver operating characteristic (ROC) curve analysis (AUC = 0.8597, 95% CI: 0.79–0.93, *P* < 0.0001), which suggests that SCARNA12 could be used for CRC diagnosis (Fig. 1h). In addition, the SCARNA12 expression level was higher in CRC cell lines (SW620, HCT116, and HT29) than in human normal colon epithelial cells (FHC) and human small intestinal epithelial cells (HIEC-6) (Fig. 1i). These data revealed that SCARNA12 may act as an oncogene in CRC.

### High expression levels of SCARNA12 were a predictor of poor prognosis in CRC patients

**Fig. 2:**
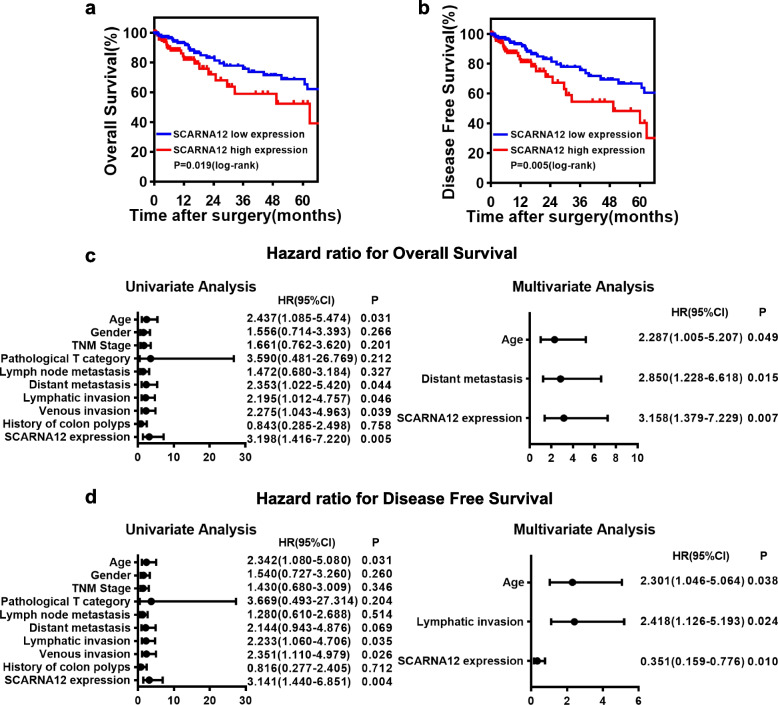
High Expression of SCARNA12 Predicted Poor Prognosis in CRC Patients. **a-b** Kaplan–Meier analysis with log-rank test assessed the impact of SCARNA12 expression levels on OS (**a**) and DFS (**b**) in CRC patients from the TCGA database. (*n* = 274, each group). **c-d** Univariate and multivariate analyses of OS (**c**) and DFS (**d**) in CRC patients (*n* = 162, each group). OS, Overall survival; DFS, Disease-Free Survival

We hypothesized that the expression of SCARNA12 was correlated with clinicopathological parameters or prognosis of patients with CRC. To address this issue, CRC patient cohorts from the TCGA database were analyzed using the χ^2^ test. The results revealed that SCARNA12 overexpression was highly correlated with a history of colonic polyps (*P* < 0.001) (Table 1). In addition, CRC patients with low SCARNA12 expression levels were identified to have longer overall survival (OS) (*P* = 0.019) and higher disease-free survival (DFS) (*P* = 0.005) than patients with high SCARNA12 expression levels using Kaplan–Meier and log-rank test (Fig. 2a, b).

**Table 1:** Correlations between SCARNA12 expression and clinicopathological parameters of CRC patients

		**SCARNA12 Expression**			
**characteristics**	**case**	**Low**	**High**	**χ**^**2**^	***P***
Sex				0.013	0.910
Female	157	78	79		
Male	157	79	78		
Age(year)				0.116	0.733
≤ 70	175	89	86		
> 70	139	68	71		
TNM stage(AJCC)				0.052	0.820
Stage I/II	178	88	90		
Stage III/IV	136	69	67		
Pathological T category				0.183	0.669
T1/T2	61	32	29		
T3/T4	253	125	128		
Lymph node metastasis				0.117	0.732
Negative	181	89	92		
Positive	133	68	65		
Distant metastasis				0.211	0.646
Negative	263	133	130		
Positive	51	24	27		
Lymphatic invasion				3.007	0.083
Negative	191	103	88		
Positive	123	54	69		
Venous invasion				0.017	0.897
Negative	233	117	116		
Positive	81	40	41		
History of colon polyps				11.504	< 0.001
Negative	214	121	93		
Positive	100	36	64		

Cutoff threshold of SCARNA12 expression is median value in all patients in this cohort

Abbreviations: *AJCC* American Joint Committee on cancer

Furthermore, the prognostic factors for OS and DFS of CRC patients were assessed by univariate and multivariate Cox proportional hazards models. As shown in Fig. 2c, d, the expression level of SCARNA12 was an independent prognostic factor for OS (HR = 3.158, 95% CI = 1.379–7.229, *P* = 0.007) and DFS (HR = 0.351, 95% CI = 0.159–0.776, *P* = 0.010). The aforementioned results revealed that SCARNA12 had potential prognostic value and could be employed as a prognostic biomarker in CRC patients.

### SCARNA12 promoted CRC cell proliferation and survival

**Fig. 3:**
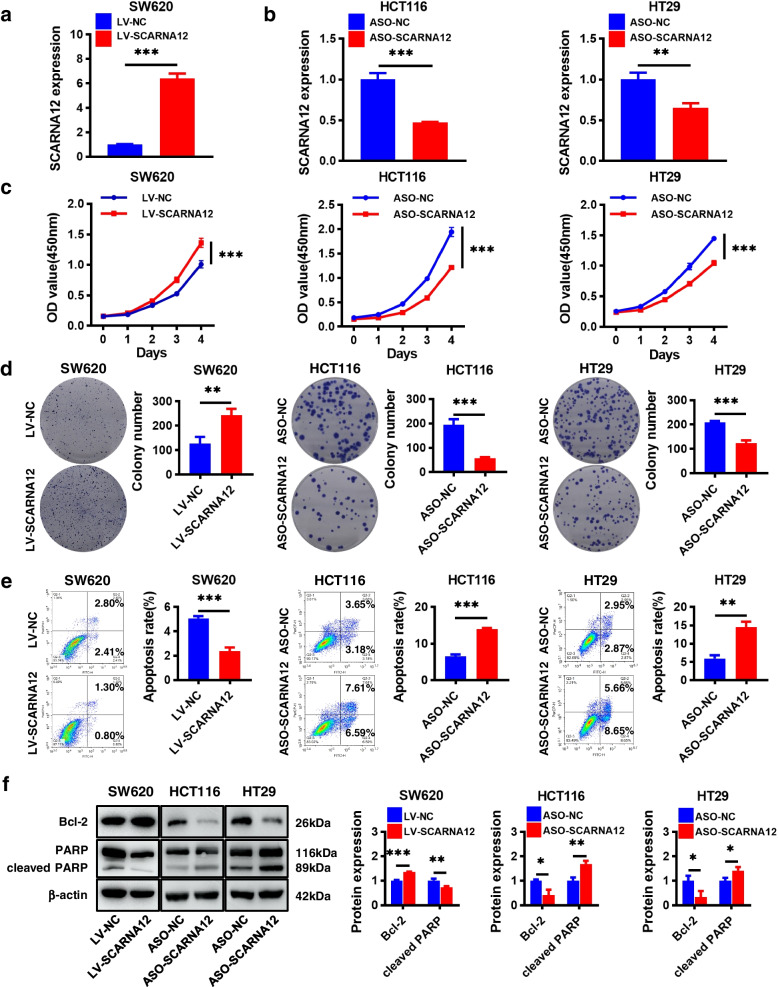
The effects of SCARNA12 on cell proliferation, colony formation, and apoptosis in CRC cells. **a-b** The levels of SCARNA12 in CRC cells infected with lentiviruses LV-SCARNA12 (**a**) or transfected with ASO-SCARNA12 (**B**). **c-d** CRC cells’ proliferative capacity assessed by CCK-8 assays (**c**) and colony formation assays (**d**). **e** The apoptotic rates of CRC cells assessed by flow cytometry experiments. **f** The expression levels of Bcl-2 and cleaved PARP proteins in CRC cells detected by Western blot. The results are presented as the mean ± SD (n ≥ 3). * *P* < 0.05, ** *P* < 0.01, *** *P* < 0.001

To investigate the potential function of SCARNA12, we predicted its secondary structure using the RNAfold web server and performed homology analysis using the UCSC Genome Browser (Fig. S2a, b). Meanwhile, HIEC-6 and SW620 cells with low endogenous SCARNA12 expression levels were infected with lentivirus overexpressing SCARNA12. HCT116 and HT29 cells were transfected with SCARNA12-targeting antisense oligonucleotide (ASO) because of high levels of SCARNA12 in the two cell lines. The SCARNA12 expression level was significantly upregulated in the LV-SCARNA12 groups of HIEC-6 and SW620 cells (Fig. S3a and Fig. 3a); meanwhile, it was remarkably downregulated in the ASO-SCARNA12 groups of HCT116 and HT29 cells (Fig. 3b). Furthermore, lentivirus infection and ASO transfection were performed to manipulate SCARNA12 expression in breast cancer cells (MCF7) and non-small cell lung cancer (NSCLC) cells (NCI-H1299; Figs. S4a and S5a).

SCARNA12 overexpression had no significant impact on cell proliferation and colony formation in HIEC-6 cells (Fig. S3b, c). However, SCARNA12 overexpression in SW620 cells accelerated cell proliferation and colony formation. Conversely, SCARNA12 knockdown in HCT116 and HT29 cells yielded contrasting results (Fig. 3c, d). Likewise, similar outcomes were observed in MCF7 and NCI-H1299 cells (Figs. S4b, c and S5b, c).

Flow cytometry analyses revealed that SCARNA12 overexpression suppressed apoptosis in SW620 cells, while SCARNA12 knockdown significantly induced apoptosis in HCT116 and HT29 cells (Fig. 3e). Western blot results indicated that SCARNA12 overexpression enhanced Bcl-2 expression and decreased cleaved-PARP levels. Conversely, SCARNA12 knockdown showed a contrasting trend (Fig. 3f). In MCF7 cells, SCARNA12 overexpression had almost no impact on cell apoptosis, while it only slightly suppressed apoptosis in NCI-H1299 cells. In both cell types, SCARNA12 overexpression did not significantly affect the expression of Bcl-2 and cleaved-PARP (Fig. S4d, e). However, SCARNA12 knockdown in MCF7 and NCI-H1299 cells significantly induced apoptosis, aligning with the observed phenotype in CRC (Fig. S5d, e).

### SCARNA12 had no effect on host gene PHB2

**Fig. 4:**
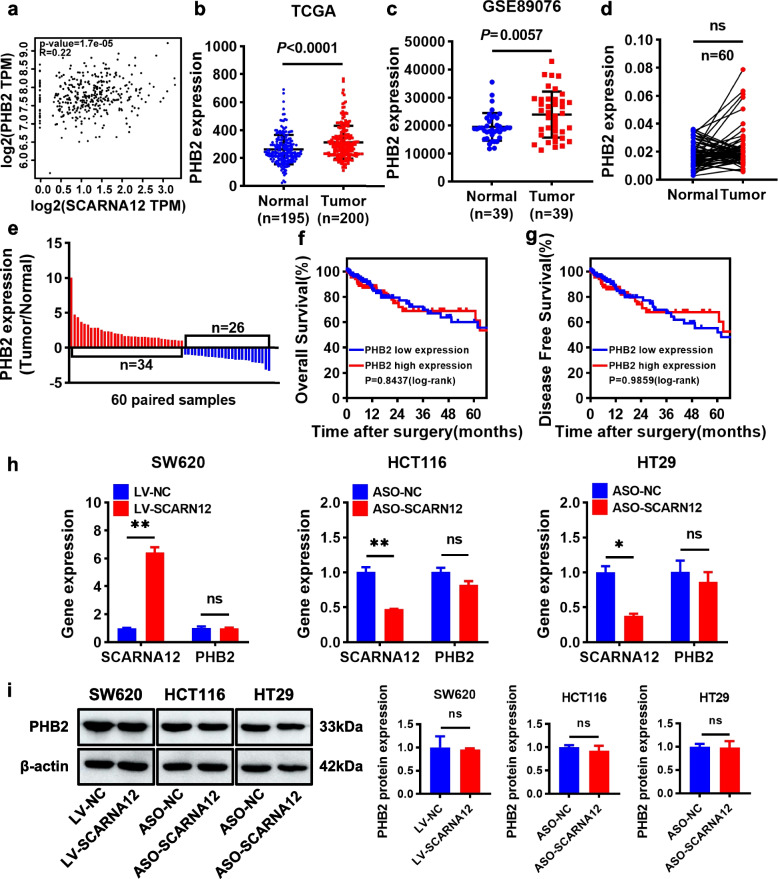
SCARNA12 had no effect on host gene PHB2. **a** The correlation between SCARNA12 and PHB2 in CRC tissues was analyzed from GEPIA website. **b-c** Relative expression of PHB2 in CRC tissues and normal tissues from TCGA database (normal = 195, tumor = 200) (**b**)**,** GEO GSE89076 dataset (*n* = 39, each group) (**c**). **d-e** The expression levels of PHB2 in 60 pairs of CRC tissues and matched para-cancer tissues were detected by qRT-PCR (**d**). Red columns indicate upregulated and the blue represent downregulated in **e**. **f-g** Kaplan–Meier analysis with log-rank test assessed the impact of PHB2 expression levels on OS (**f**) and DFS (**g**) in CRC patients from the TCGA database (PHB2 low expression = 223, PHB2 high expression = 222). **h-i** Effect of SCARNA12 on the expression of PHB2 mRNA **(h)** and protein** (i)**. The results are presented as the mean ± SD (*n* = 3). ns, no significance; * *P* < 0.05, ** P < 0.01

In mammals, snoRNA exists in the intron region of the host gene without independent promoter and relies on transcription and splicing of its host gene for expression. Therefore, the host gene can regulate the abundance of snoRNA expression [32]. Conversely, snoRNA can also alter the final output fate of host gene by regulating alternative splicing, which in turn affects its expression [33]. In view of the bidirectional relationship between snoRNA and the host, we speculated that SCARNA12 exerts its function by influencing host PHB2 expression. GEPIA analysis was performed for validation, and the results revealed that SCARNA12 was positively correlated with PHB2 (Fig. 4a). Furthermore, TCGA and GEO databases (GSE89076) were employed to analyze the expression level of PHB2 in CRC. As shown in Fig. 4b, c, PHB2 was significantly upregulated in CRC tissues compared with adjacent normal tissues. However, PHB2 was highly expressed in only 57% of the CRC clinical samples (Fig. 4d, e). Meanwhile, the PHB2 expression level did not affect OS (*P* = 0.8437, Fig. 4f) or DFS (*P* = 0.9859, Fig. 4g), which was inconsistent with the role of SCARNA12. In addition, we found that after the overexpression or knockdown of SCARNA12, the mRNA and protein expression levels of PHB2 did not change (Fig. 4h, i), which further proved that PHB2 did not mediate the regulatory effect of SCARNA12 on the biological effects of CRC cells.

### SCARNA12 promoted CRC cell proliferation and survival by activating the PI3K/AKT pathway

**Fig. 5:**
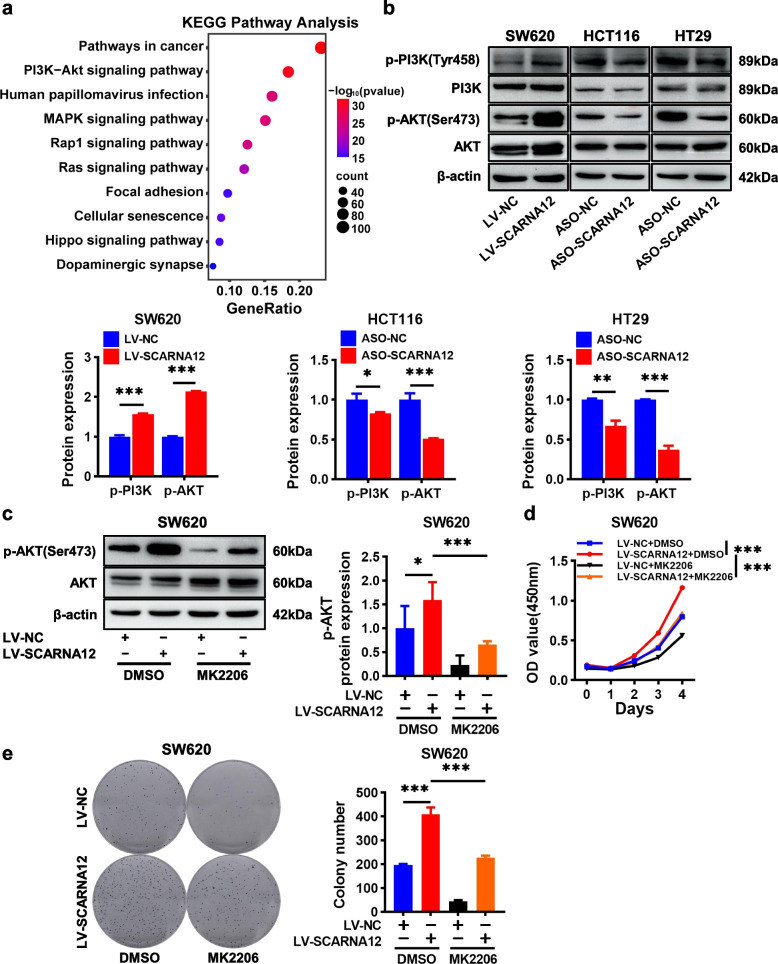
Overexpressing of SCARNA12 facilitated CRC cells growth via activating PI3K/AKT pathway. **a** KEGG pathway enrichment analysis of differentially expressed genes after SCARNA12 knockdown. **b** SCARNA12 activates the PI3K/AKT pathway. **c** Effect of AKT inhibitor MK2206 on p-AKT protein expression level in SW620 cells. **d-e** MK2206 reversed the SCARNA12-induced biological effects in SW620 cells. The results are presented as the mean ± SD (*n* ≥ 3). * *P* < 0.05, ** *P* < 0.01, *** *P* < 0.001

To elucidate the potential mechanism of SCARNA12 in CRC, RNA-sequencing was performed on HCT116 cells transfected with ASO-NC and ASO-SCARNA12, respectively. The knockdown efficiency of SCARNA12 was detected by qRT-PCR (Fig. S6a). Using thresholds of log2 fold change (FC) > 1 or <  − 1, along with a p-value of < 0.05, we identified 1515 differentially expressed genes. Among them, 450 genes showed downregulated expression and 1065 genes showed significantly upregulated expression (Fig. S6b). The top 10 items of gene ontology (GO) enrichment analysis revealed the effects of SCARNA12 alteration in CRC on biological processes, cell compositions, and molecular functions (Fig. S6c-e). Additionally, we performed enrichment analysis of the Kyoto Encyclopedia of Genes and Genomes (KEGG) signaling pathway and found that the PI3K/AKT pathway, which is closely linked with tumor progression, was markedly enriched (Fig. 5a). These findings suggested that SCARNA12 may be a critical regulator of the PI3K/AKT pathway. In order to ensure data accuracy, several differentially expressed genes involved in PI3K/AKT pathway were heat mapped (Fig. S6f) and verified by qRT-PCR. SCARNA12 knockdown notably reduced the basal expression levels of EGFR, CCND1, FGF9, and PIK3R3 in HCT116 cells (Fig. S6g).

The PI3K/AKT pathway is abnormally activated in tumors owing to phosphorylation at specific sites, resulting in uncontrolled cell proliferation and survival. In order to analyze whether this pathway is affected by SCARNA12 levels, western blot assay was used for further verification. First, SCARNA12 overexpression in HIEC-6 cells had no effect on the PI3K/AKT pathway, which may be the reason why SCARNA12 did not promote the proliferation of HIEC-6 cells (Fig. S7). Second, the overexpression of SCARNA12 in SW620 cells enhanced the phosphorylation levels of PI3K and AKT at Tyr458 and Ser473, respectively; and the knockdown of SCARNA12 in HCT116 and HT29 cells reduced their phosphorylation levels. Notably, the expression level of SCARNA12 did not affect the overall levels of PI3K and AKT (Fig. 5b). Lastly, we checked whether the inhibition of p-AKT expression affects the functions of SCARNA12 in CRC cells. The data showed that the AKT inhibitor (MK-2206) significantly inhibited the expression of p-AKT and reversed the proliferative and colony formation promotion effect of SCARNA12 on SW620 cells (Fig. 5c-e). Our results revealed that SCARNA12 regulated the malignant biological behaviors of CRC cells, attributable to activating the PI3K/AKT pathway, at least in part.

### SCARNA12 promoted CRC xenograft tumor growth

**Fig. 6:**
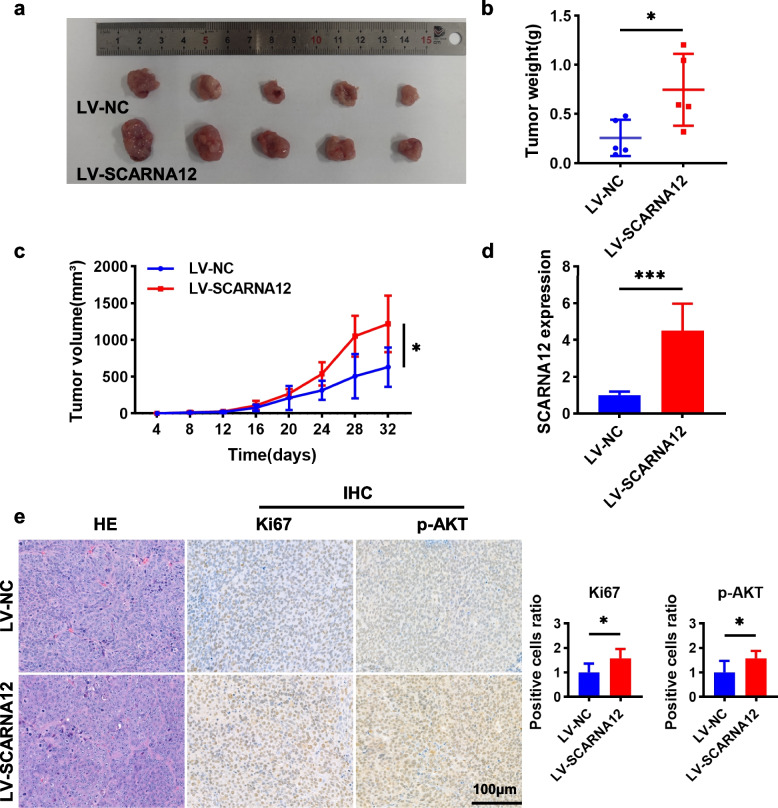
Overexpression of SCARNA12 promoted xenograft tumor growth. **a** Subcutaneous injection of SW620/LV-NC and SW620/LV-SCARNA12 cells into nude mice for xenograft tumor assay (*n* = 5, each group). **b-c** Xenograft tumors weights (**b**) and volumes growth curve (**c**) were measured every 4 days. **d** The expression levels of SCARNA12 in xenograft tumors. **e** The xenograft tumor tissue sections were stained with H&E, and the expression levels of Ki67 and p-AKT were assessed by IHC staining to determine the ratio of positive cells. The results are presented as the mean ± SD (*n* = 5). * *P* < 0.05; *** *P* < 0.001

A xenograft tumor model was established to further analyze the biological functions of SCARNA12 in vivo. SW620 cell lines stably infected with LV-NC or LV-SCARNA12 were inoculated subcutaneously into nude mice to develop implant-tumor. Figure 6a, b revealed that the size and weight of transplanted tumors in nude mice overexpressing SCARNA12 were significantly increased, along with the volume of transplanted tumors (Fig. 6c). Moreover, SCARNA12 was highly expressed in xenograft tumors infected with LV-SCARNA12 (Fig. 6d). Besides, immunohistochemical (IHC) analysis revealed that the positive rates of Ki67 and p-AKT were notably elevated in xenograft tumor tissues overexpressing SCARNA12 (Fig. 6e). The above results showed that SCARNA12 promoted tumor growth and activated the PI3K/AKT pathway in vivo, which was consistent with our results in vitro.

## Discussion

Since first identified, about 30 scaRNAs have been described as guide RNAs responsible for posttranscriptional modifications of target RNAs, occasionally as telomerase RNA to elongate telomeres [34]. Recent studies led researchers to focus on the function of scaRNAs in carcinogenesis, although most of them are poorly characterized. In this study, SCARNA12 was highly expressed in multiple cancers, including CRC. The expression levels of SCARNA12 were closely related to a history of polyps and poor prognosis in CRC patients. The abnormal expression of SCARNA12 affects the proliferation, survival and apoptosis of CRC cells. Mechanically, SCARNA12 enhances the proliferation and tumorigenicity of CRC cells by activating the PI3K/AKT signaling pathway, rather than regulating the expression of its host gene PHB2.

Growing evidence suggests that snoRNA is involved in tumor development as an oncogene or tumor suppressor gene. For example, SNORA21, SNORA24, and SNORA42 are upregulated in CRC and promote tumor growth both in *vivo* and in *vitro*; SNORD1C maintains the stemness of CRC cells and resistance to the chemotherapeutic drug 5-FU by activating the Wnt pathway. High expressed levels of these genes all lead to poor prognoses in CRC patients [22–24, 35]. However, SNORD44 is downregulated in CRC and has an anti-tumor effect [25]. In 2019, Peng‐Fei Zhang et al. found that SCARNA2 increased CRC cell resistance to 5-FU and oxaliplatin and indicated poor outcomes for patients [27]. It is the only scaRNA gene identified in the carcinogenesis of CRC. These lines of evidence have encouraged us to explore the role of SCARNA12 in the pathological process of CRC to understand its value in clinical settings. Bioinformatics analyses have previously shown that SCARNA12 could be a diagnostic biomarker for cervical cancer [31]. In this study, data showed that overexpression of SCARNA12 promoted the proliferation and tumorigenicity of CRC cells; conversely, the knockdown of SCARNA12 significantly inhibited proliferation and colony formation in CRC cells, and induced apoptosis. In addition, the gain or loss of SCARNA12 affected cell proliferation and colony formation in MCF-7 and NCI-H1299 cells, which suggests that SCARNA12 might exert a broad-spectrum proliferation promotion effect in different tumors. The scaRNA family consists of various members, including the RNA component of telomerase (TERC) [34, 36], which is believed to play a crucial role in regulating telomerase activity and telomere maintenance [37, 38]. Therefore, we have a tempting speculation that the abnormal expression of SCARNA12 may let CRC cells gain the ability of unrestricted proliferation by elongating telomeres. Regrettably, we have not yet investigated the correlation between SCARNA12 and telomere lengthening.

Evidence has shown that snoRNA host genes also have important regulatory effects on tumor biological functions. For example, GAS5 is the host gene of SNORD44, both of which are downregulated in CRC and play a synergistic role in inhibiting cell proliferation and inducing cell apoptosis [25]. SNHG25, another non-coding SNORA50C host gene, is highly expressed in neuroblastomas (NBs), and the knockdown of SNHG25 inhibits the proliferation, migration, and invasion of NB cells [32]. However, this is not always the case. Although CCNB1IP1 serves as the host gene for SNORD126, Fang et al. revealed that SNORD126 was not significantly correlated with CCNB1IP1 expression levels, and SNORD126 functioned independently of CCNB1IP1 in CRC cells [39]. To investigate the relationship between SCARNA12 and its host gene PHB2, we examined clinical samples from CRC patients and found that SCARNA12 and PHB2 were highly expressed in 88% and 57% of CRC tissues, respectively. It has been reported that PHB2 enhances the proliferation and tumorigenicity of CRC cells [40], promotes the migration and invasion of lung cancer cells [41], and affects tamoxifen resistance in breast cancer cells [42], suggesting that PHB2 dysregulation may play multiple functions in tumors. In this study, our data showed that the overexpression or knockdown of SCARNA12 did not affect the mRNA and protein levels of PHB2, indicating that PHB2 was not involved in SCARNA12 promoting the biological effect of CRC cells.

The PI3K/AKT pathway, an important intracellular signaling pathway in mammals, is physiologically activated by insulin, growth factors, and cytokines, and participates in multiple biological processes, including cell proliferation, survival, metastasis, and metabolism [43]. It is abnormally activated in many types of cancers, such as, colorectal [44], lung [45], prostate [46], stomach [47, 48], ovarian [49, 50], and breast [51, 52]. In recent years, a few studies have reported on the effect of snoRNAs on the PI3K/AKT pathway. Fang et al. discovered that SNORD126 activates the PI3K/AKT pathway by upregulating FGFR2 and inducing the phosphorylation of AKT on Ser473 in CRC and human hepatocellular carcinoma (HCC) [39]. Another case is ACA11, proven by Wu et al. [53]. However, the interaction between scaRNAs and the PI3K/AKT pathway has not been reported. In this study, we revealed that SCARNA12 targeted the PI3K/AKT pathway and positively regulated the expression of p-PI3K (Tyr458) and p-AKT (Ser473), which generally recognized as hallmarks of PI3K/AKT pathway activation. Moreover, MK-2206 blocking AKT activity greatly attenuated the cancer-promoting effect of SCARNA12 in CRC cells, supporting the theory that SCARNA12 promotes the proliferation of CRC cells by activating the PI3K/AKT signaling pathway.

Although we are the first to demonstrate that SCARNA12 promotes CRC progression by activating the PI3K/AKT pathway, several limitations are still associated with this study: (1) the detailed mechanism of phosphorylating PI3K/AKT by which SCARNA12 needs to be explored further; (2) more clinical data and experimental studies are required to elucidate the specific function of SCARNA12 in CRC.

In summary, this study confirms that SCARNA12 is upregulated in various cancers, including CRC. It was also proven that SCARNA12 can function as a novel oncogene during the pathological process of CRC and promote CRC progression by activating the PI3K/AKT pathway. Our study illustrated the underlying clinical value of SCARNA12 as a risk diagnostic biomarker in patients with CRC.

## Materials and methods

### Bioinformatics analysis

SCARNA12 expression in CRC patient samples was analyzed using the TCGA (https://tcgadata.nci.nih.gov/tcga), GEO (https://www.ncbi.nlm.nih.gov/gds), UALCAN (https://ualcan.path.uab.edu), and GEPIA (http://gepia2.cancer-pku.cn) databases. The relevance between SCARNA12 or PHB2 expression and OS and DFS was analyzed using data downloaded from TCGA for CRC patients. Additionally, the correlation between SCARNA12 expression and various clinicopathological parameters was evaluated using the data as well. The secondary structure of SCARNA12 was predicted using the RNAfold web server (http://rna.tbi.univie.ac.at//cgi-bin/RNAWebSuite/RNAfold.cgi). SCARNA12 homology analysis was performed using the UCSC Genome Browser (http://genome.ucsc.edu). The Database for Annotation, Visualization and Integrated Discovery (DAVID, https://david.ncifcrf.gov/home.jsp) was used to perform GO and KEGG analyses, to predict the function of SCARNA12.

### Patients and samples

Sixty pairs of CRC tissue and adjacent normal mucosal tissue specimens were collected from Liaoning Cancer Hospital. The Ethics Committee on Human Investigation of Liaoning Cancer Hospital (20,190,970) approved the use of the specimens, and each patient provided informed consent for this study.

### Cell culture

FHC was purchased from the American Type Culture Collection (ATCC, Manassas, Virginia), HIEC-6 was preserved by our laboratory for years, and CRC cell lines (SW620, HT29, and HCT116) were purchased from GeneChem (Shanghai, China). MCF7 and NCI-H1299 were purchased from the Chinese National Infrastructure of Cell Line Resource (NICR; Beijing, China). The culture conditions of all the cells were the same as those described in previously published study [23, 54, 55].

### RNA extraction and quantitative real time-PCR (qRT-PCR)

TRIzol reagent (Sigma, USA) was used to extract total RNA from tissue samples or cells. The PrimeScript™ RT reagent Kit (Takara, Shiga, Japan) was used to reverse-transcribe RNA into cDNA. In a Bio-Rad CFX96 system, qRT-PCR was carried out using the iTaq Universal SYBR Green Supermix (BioRad, USA) and specific primers (Table S1). Finally, GAPDH or U6 was used as an internal control to determine gene expression levels based on the 2^−∆∆Ct^ method.

### Cell transfection

SW620 cells were infected with lentivirus overexpressing SCARNA12 and negative control lentivirus (GeneChem, Shanghai, China). Then, 72 h after the lentivirus infection, puromycin (2 μg/mL) was used to screen the cells. RiboBio (Guangzhou, China) designed and synthesized the ASO for targeting SCARNA12. The oligonucleotide sequence of ASO-SCARNA12 was 5´- CTAGTTCCTGGCAGCAGCCT -3´. ASO was used to transfect HCT116 and HT29 cells in accordance with the manufacturer’s instructions.

### Cell proliferation assay

The ability of cells to undergo proliferation was measured using Cell Counting Kit-8 (Dojindo, Kumamoto, Japan) in accordance with the manufacturer’s instructions. Cells were seeded in 100 μL of medium containing 2 × 10^3^ cells/well on 96-well plates; meanwhile, five replicates were set in each group. Then, 10% CCK-8 reagent was added to each well and incubation was continued at 37℃ for 2 h. The absorbance was measured using a microplate reader (BIO-RAD, 170–6750) at 450 nm.

### Colony formation assay

Eight hundred cells were seeded into 6-well plates and cultured for 2 weeks, while changing the medium every 4 days. Cells were fixed with methanol for 20 min and then stained using Giemsa for 30 min at room temperature. The total number of colonies in each well was counted in an unbiased manner.

### Cell apoptosis analysis

Cells from different groups were stained with the Annexin V-FITC Apoptosis Detection kit (DOJINDO). Flow cytometry (ACEA Bio, San Diego, California) was used to detect the percentages of apoptosis cells. Each experiment was performed in triplicate.

### Western blot

Cells were lysed in RIPA buffer supplemented with phosphatase and protease inhibitors (Roche). The BCA Protein Assay Kit (Beyotime, P0010) was applied for quantifying protein concentrations. Equal amounts of protein (30 μg) were separated by 10% SDS–polyacrylamide gel electrophoresis (SDS-PAGE) and transferred to nitrocellulose membranes. Subsequently, 5% fat-free milk powder was used to block the membranes for 2 h, and the membranes were incubated overnight with primary antibodies of PI3K (#4292, CST, 1:1000), p-PI3K (#4228, CST, 1:1000), AKT (#4691, CST, 1:1000), p-AKT (#4060, CST, 1:1000), PARP (#9542, CST, 1:1000), Bcl-2 (#YM3041, ImmunoWay, 1:2000), and β-actin (60,009–1-Ig, Proteintech, 1:5000) at 4℃. The membranes were washed three times with TBS-Tween 20 (TBS-T), and then incubated for 2 h with secondary antibodies of rabbit IgG (#5220–0336, KPL, 1:5000) or mouse IgG (#5220–0341, KPL, 1:5000). Finally, the protein bands were visualized using the ECL Kit (Thermo Fisher Scientific).

### Nude mice tumor xenograft assay

Xenograft models were established using 5-week-old male BALB/c nude mice (Beijing Viton Lihua, China). Subsequently, the nude mice were randomly divided into two groups (LV-NC, LV-SCARNA12, n = 5). SW620 cells (about 5 × 10^6^ cells per site in 100 μL PBS) infected with LV-SCARNA12 or LV-NC were subcutaneously injected into mice. The volumes of the xenografts were measured with vernier calipers every 4 days. After 30 days, subcutaneous xenografts of different groups were collected, photographed, and weighed. All animal experiments were carried out in accordance with the ethical regulations of the Ethics Committee of the Academy of Military Medical Science (IACUC-DWZX-2022–832, Beijing, China).

### Hematoxylin and eosin and immunohistochemistry

Hematoxylin and eosin (H&E) and IHC staining were performed as described previously [23]. The antibodies used were Ki-67 (Cat. No. ab15580, Abcam, 1:2000) and p-AKT (Cat. No. A11016, ABclonal, 1:100). The color development reaction was achieved using 3,3ʹ-diaminobenzidine tetrahydrochloride (DAB).

### Statistical analyses

All experiments were performed in triplicate. All data were represented as mean ± SD values. Kaplan–Meier method and log-rank test were used for survival analyses. Chi-square (χ^2^) test was used to evaluate the correlation of SCARNA12 expression with clinicopathological parameters in CRC patients. The independent prognostic factors related to survival were identified by the Cox proportional hazards regression model. The data of different groups were analyzed by the two-tailed Student’s t-test. SPSS 26.0 and GraphPad Prism 9.0 software were used to perform all statistical analyses. *P* < 0.05 was considered statistically significant.

### Supplementary Information

**Additional file 1: Fig. S1. **SCARNA12 was highly expressed in a variety of tumors. **Fig. S2.** Secondary structure and homology analysis of SCARNA12. a The secondary structure of SCARNA12 was predicted using RNAfold web server. b The homology analysis of SCARNA12 performed using the UCSC Genome Browser. **Fig. S3.** The effects of SCARNA12 on cell proliferation and colony formation in HIEC-6. a The levels of SCARNA12 in HIEC-6 infected with lentiviruses LV-SCARNA12. b The proliferative capacity of HIEC-6 assessed by CCK-8 assays. c The colony formation assays of HIEC-6. The results are presented as the mean ± SD (*n* ≥ 3). ns, no significance; *** *P* < 0.001. **Fig. S4.** The effects of overexpressing SCARNA12 on cell proliferation, colony formation, and apoptosis in breast cancer and NSCLC cells. a The levels of SCARNA12 in breast cancer cells (MCF7) and NSCLC cells (NCI-H1299) infected with lentiviruses LV-SCARNA12. b The proliferative capacity of MCF7 and NCI-H1299 assessed by CCK-8 assays. c The colony formation assays of MCF7 and NCI-H129. d The apoptotic rates of MCF7 and NCI-H1299 assessed by flow cytometry experiments. e The expression levels of Bcl-2 and cleaved PARP proteins in MCF7 and NCI-H1299 detected by Western blot. The results are presented as the mean ± SD (*n* ≥ 3). ns, no significance; * *P* < 0.05, *** *P* < 0.001. **Fig. S5.** The impact of SCARNA12 knockdown on cell proliferation, colony formation, and apoptosis in breast cancer and NSCLC cells. a The levels of SCARNA12 in MCF7 and NCI-H1299 transfected with ASO-SCARNA12. b The proliferative capacity of MCF7 and NCI-H1299 assessed by CCK-8 assays. c The colony formation assays of MCF7 and NCI-H129. d The apoptotic rates of MCF7 and NCI-H1299 assessed by flow cytometry experiments. e The expression levels of Bcl-2 and cleaved PARP proteins in MCF7 and NCI-H1299 detected by Western blot. The results are presented as the mean ± SD (*n* ≥ 3). * *P* < 0.05, ** *P* < 0.01, *** *P* < 0.001. **Fig. S6.** Exploring the underlying biological mechanisms involved in SCARNA12. a The knockdown efficiency of SCARNA12 in HCT116. b Volcano plot revealed the differentially expressed genes obtained from RNA-seq analysis of HCT116/ASO-NC and HCT116/ASO-SCARNA12 groups. c-e Top 10 strikingly enriched GO annotations for cellular component (c), molecular function (d), biological process (e). f The heat map presented representative differential genes determined by RNA-seq analysis in the PI3K/AKT signaling pathway. g The levels of indicated target genes verified by qRT-PCR. The results are presented as the mean ± SD (*n* = 3). ***P < 0.001. **Fig. S7.** The proteins expression of PI3K/AKT pathway in HIEC-6. The results are presented as the mean ± SD (*n* = 3). ns, no significance. **Table S1.** Primer sequences used in this study

## Data Availability

The data supporting this study are available from the corresponding author upon reasonable request.
