# Supplementary material for: Oncogene SCARNA12 as a potential diagnostic biomarker for colorectal cancer
Source: Mol Biomed. 2023 Nov 1;4:37. doi: 10.1186/s43556-023-00147-x (PMC10618143; doi:10.1186/s43556-023-00147-x)
Supplement: Supplementary file 1 — Additional file 1: Fig. S1. SCARNA12 was highly expressed in a variety of tumors. Fig. S2. Secondary structure and homology analysis of SCARNA12. a The secondary structure of SCARNA12 was predicted using RNAfold web server. b The homology analysis of SCARNA12 performed using the UCSC Genome Browser. Fig. S3. The effects of SCARNA12 on cell proliferation and colony formation in HIEC-6. a The levels of SCARNA12 in HIEC-6 infected with lentiviruses LV-SCARNA12. b The proliferative capacity of HIEC-6 assessed by CCK-8 assays. c The colony formation assays of HIEC-6. The results are presented as the mean ± SD (n ≥ 3). ns, no significance; *** P < 0.001. Fig. S4. The effects of overexpressing SCARNA12 on cell proliferation, colony formation, and apoptosis in breast cancer and NSCLC cells. a The levels of SCARNA12 in breast cancer cells (MCF7) and NSCLC cells (NCI-H1299) infected with lentiviruses LV-SCARNA12. b The proliferative capacity of MCF7 and NCI-H1299 assessed by CCK-8 assays. c The colony formation assays of MCF7 and NCI-H129. d The apoptotic rates of MCF7 and NCI-H1299 assessed by flow cytometry experiments. e The expression levels of Bcl-2 and cleaved PARP proteins in MCF7 and NCI-H1299 detected by Western blot. The results are presented as the mean ± SD (n ≥ 3). ns, no significance; * P < 0.05, *** P < 0.001. Fig. S5. The impact of SCARNA12 knockdown on cell proliferation, colony formation, and apoptosis in breast cancer and NSCLC cells. a The levels of SCARNA12 in MCF7 and NCI-H1299 transfected with ASO-SCARNA12. b The proliferative capacity of MCF7 and NCI-H1299 assessed by CCK-8 assays. c The colony formation assays of MCF7 and NCI-H129. d The apoptotic rates of MCF7 and NCI-H1299 assessed by flow cytometry experiments. e The expression levels of Bcl-2 and cleaved PARP proteins in MCF7 and NCI-H1299 detected by Western blot. The results are presented as the mean ± SD (n ≥ 3). * P < 0.05, ** P < 0.01, *** P < 0.001. Fig. S6. Exploring the underlying biologic [file 43556_2023_147_MOESM1_ESM.docx]

**Oncogene SCARNA12 as a potential diagnostic biomarker for colorectal cancer**

**Hong Zhang^1,2#^, Xin Liu^2#^, Wencheng Zhang^2^, Jiarong Deng^1,2^, Chuxian Lin^2^, Zhenhua Qi^2^, Yaqiong Li^2^, Yongqing Gu^2^, Qi Wang^2^**^*^**, Liping Shen^2^**^*^**, Zhidong Wang^1,2^**^*^

**^#^**Hong Zhang and Xin Liu contributed equally to this study and share the first authorship.

*Correspondence:

Zhidong Wang

wangzdlab@126.com, wangzhidong1977@126.com

Liping Shen

shen_2011yan@sina.com

Qi Wang

wqi619@126.com

^1^ Graduate Collaborative Training Base of Academy of Military Sciences, Hengyang Medical School, University of South China, Hengyang, Hunan, 421001, China

^2^ Department of Radiobiology, Beijing Key Laboratory for Radiobiology, Beijing Institute of Radiation Medicine, Beijing, 100039, China


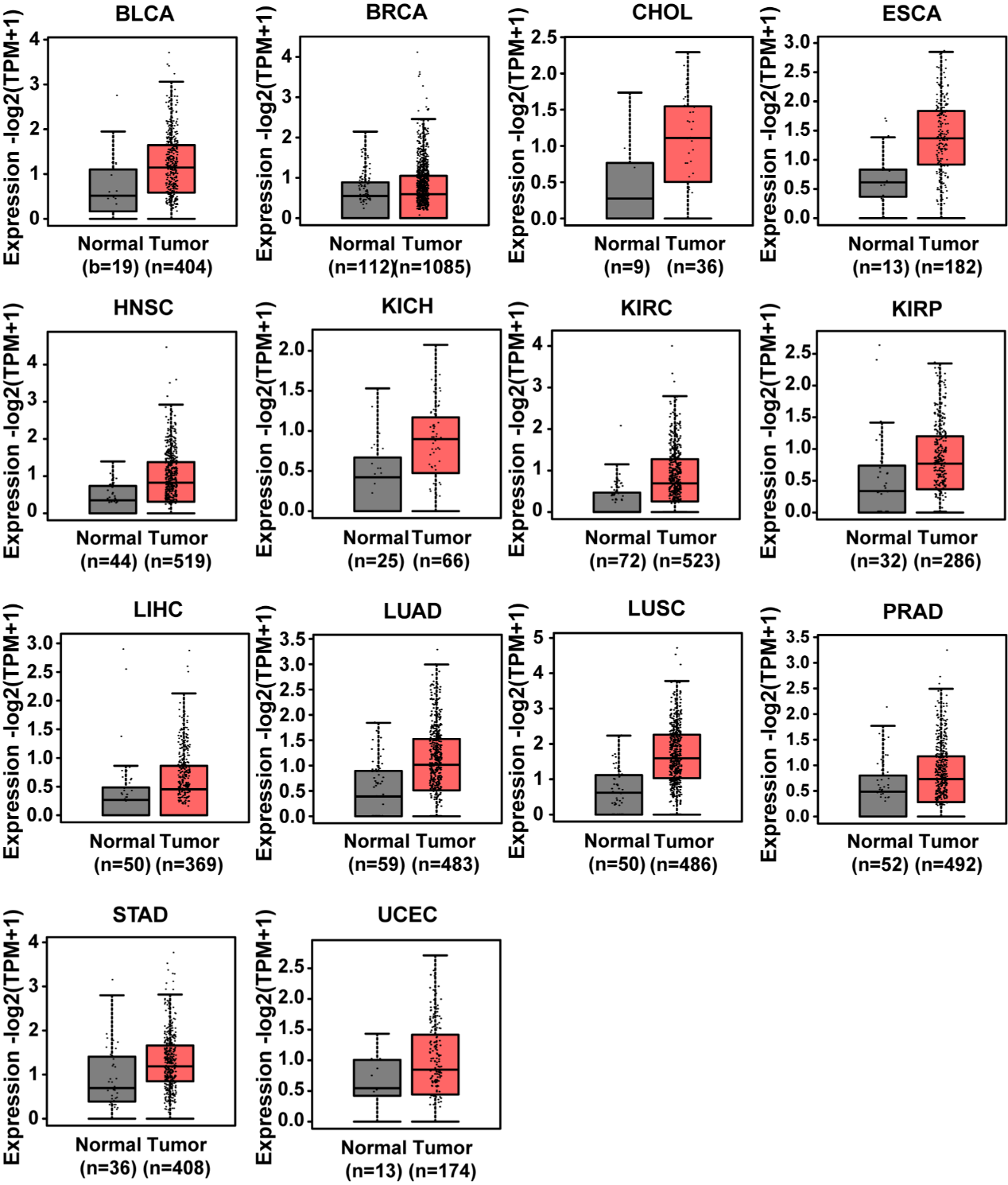
**Fig. S1** SCARNA12 was highly expressed in a variety of tumors


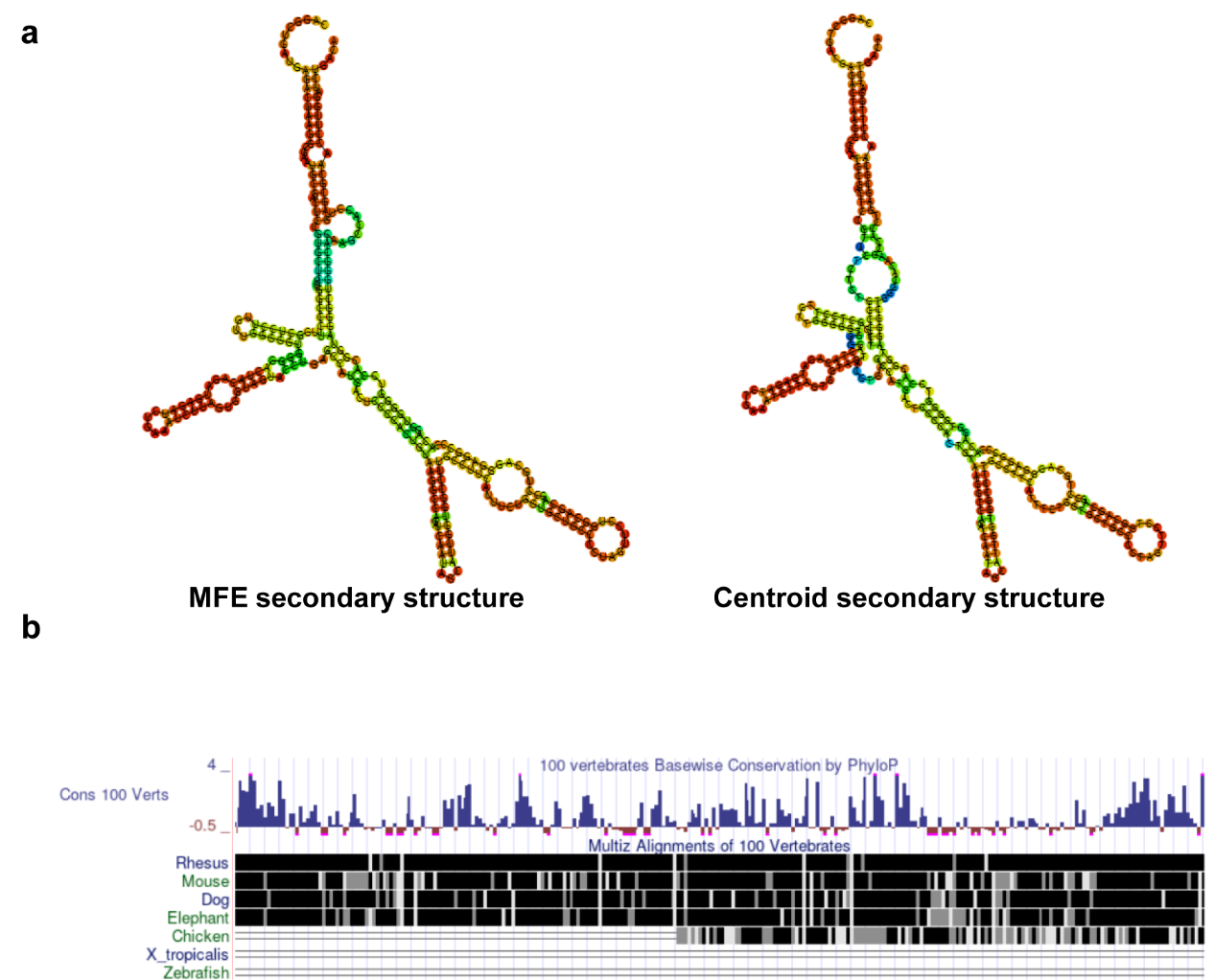


**Fig. S2** Secondary structure and homology analysis of SCARNA12. **a** The secondary structure of SCARNA12 was predicted using RNAfold web server. **b** The homology analysis of SCARNA12 performed using the UCSC Genome Browser


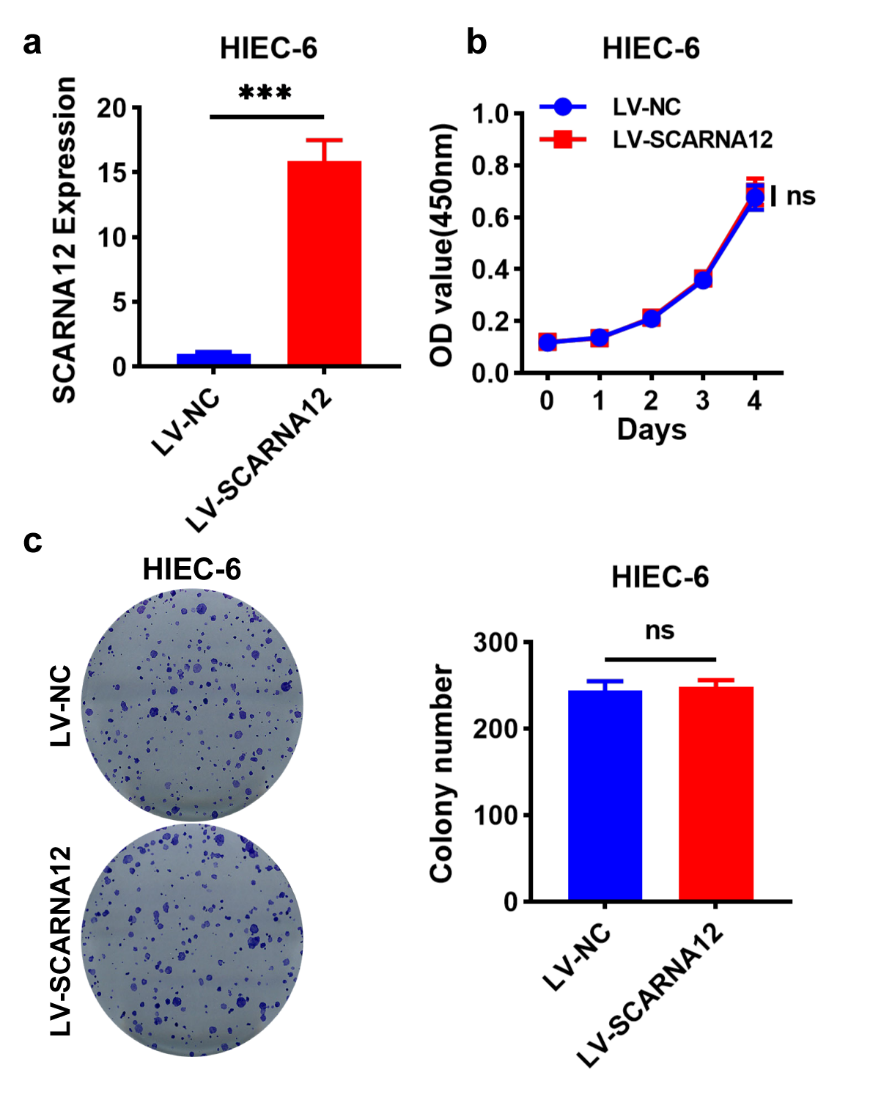


**Fig. S3** The effects of SCARNA12 on cell proliferation and colony formation in HIEC-6. **a** The levels of SCARNA12 in HIEC-6 infected with lentiviruses LV-SCARNA12. **b** The proliferative capacity of HIEC-6 assessed by CCK-8 assays. **c** The colony formation assays of HIEC-6. The results are presented as the mean ± SD (n ≥ 3). ns, no significance; *** *P* < 0.001


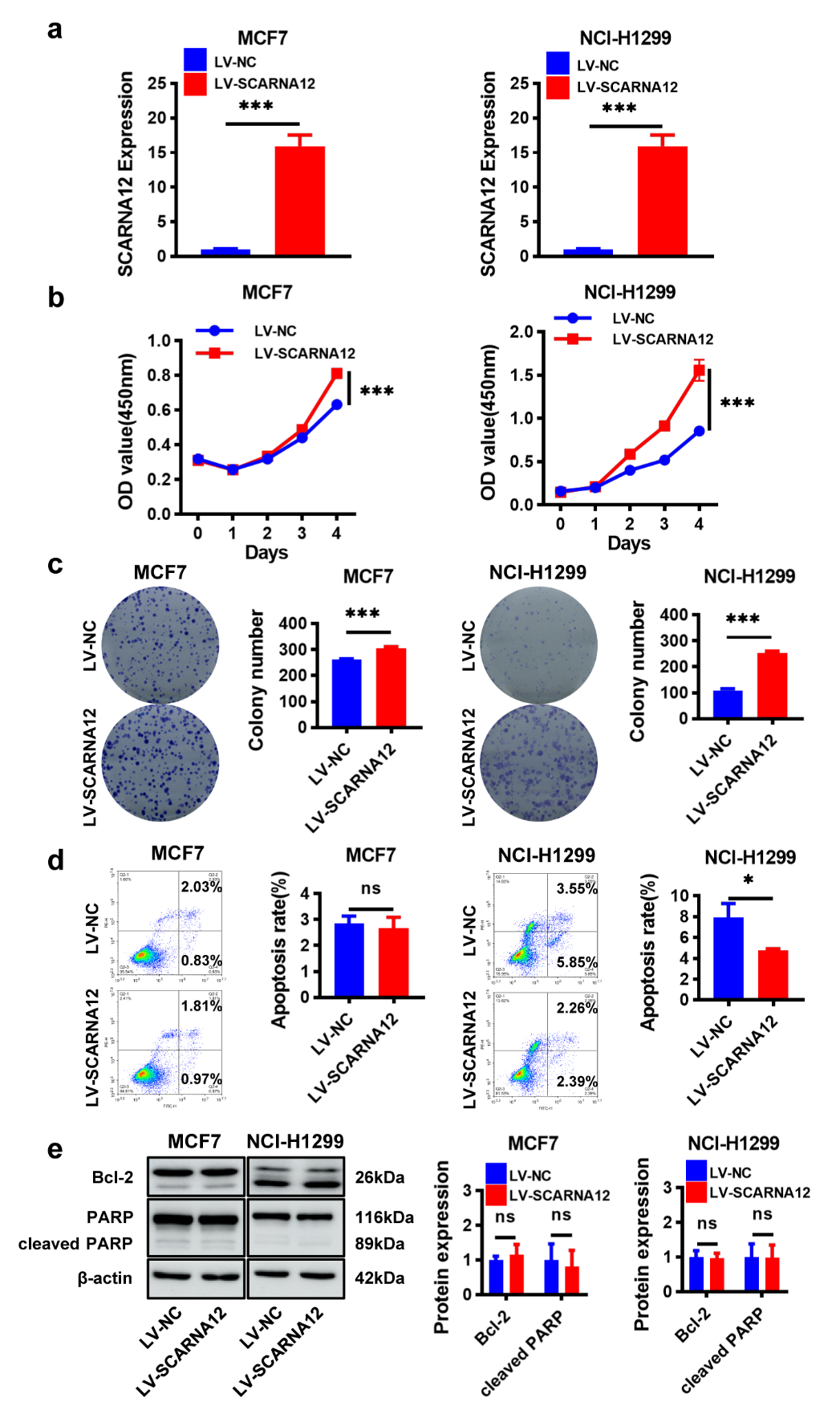


**Fig. S4** The effects of overexpressing SCARNA12 on cell proliferation, colony formation, and apoptosis in breast cancer and NSCLC cells. **a** The levels of SCARNA12 in breast cancer cells (MCF7) and NSCLC cells (NCI-H1299) infected with lentiviruses LV-SCARNA12. **b** The proliferative capacity of MCF7 and NCI-H1299 assessed by CCK-8 assays. **c** The colony formation assays of MCF7 and NCI-H129. **d** The apoptotic rates of MCF7 and NCI-H1299 assessed by flow cytometry experiments. **e** The expression levels of Bcl-2 and cleaved PARP proteins in MCF7 and NCI-H1299 detected by Western blot. The results are presented as the mean ± SD (n ≥ 3). ns, no significance; * *P* < 0.05, *** *P* < 0.001


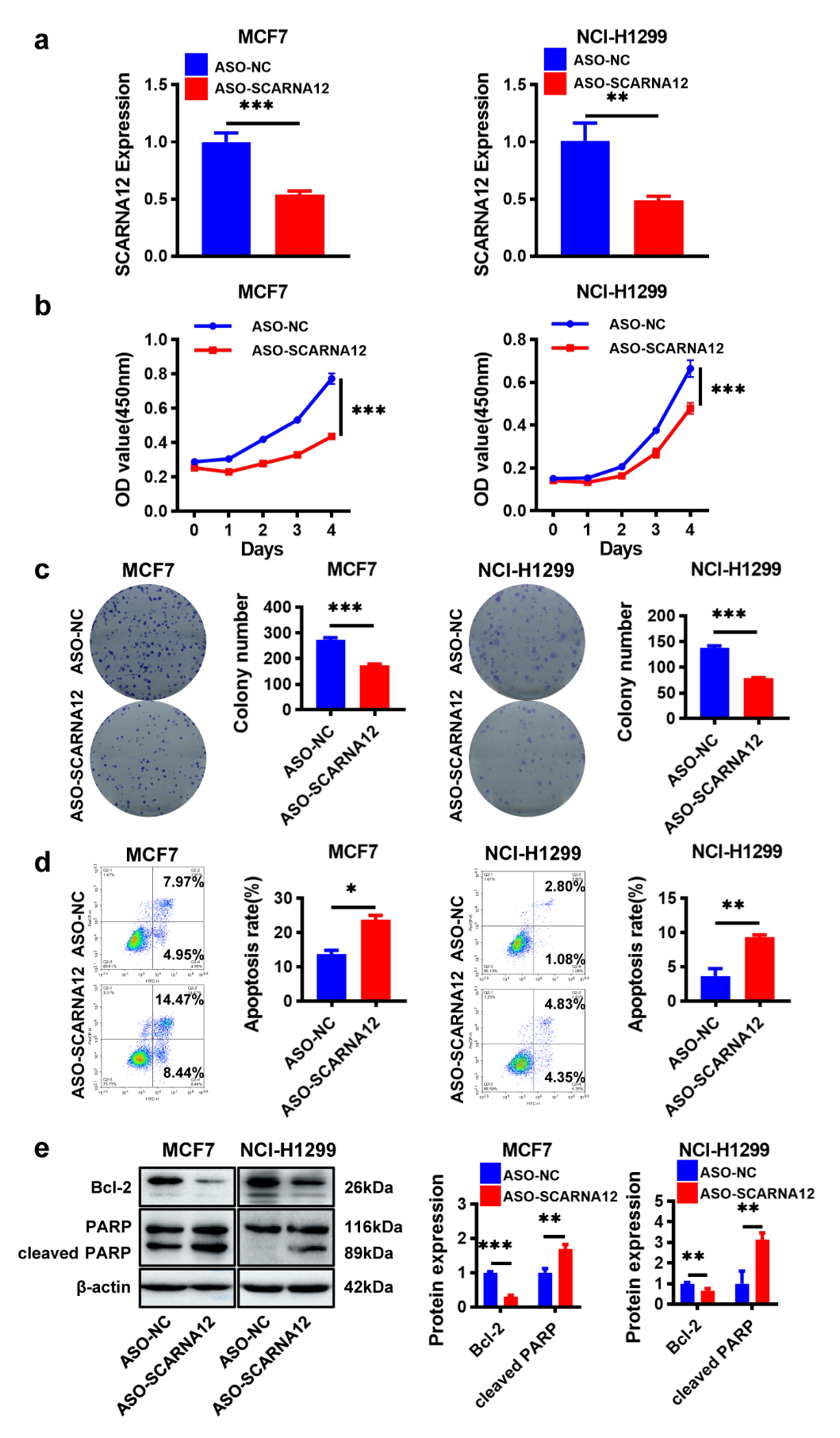


**Fig. S5** The impact of SCARNA12 knockdown on cell proliferation, colony formation, and apoptosis in breast cancer and NSCLC cells. **a** The levels of SCARNA12 in MCF7 and NCI-H1299 transfected with ASO-SCARNA12. **b** The proliferative capacity of MCF7 and NCI-H1299 assessed by CCK-8 assays. **c** The colony formation assays of MCF7 and NCI-H129. **d** The apoptotic rates of MCF7 and NCI-H1299 assessed by flow cytometry experiments. **e** The expression levels of Bcl-2 and cleaved PARP proteins in MCF7 and NCI-H1299 detected by Western blot. The results are presented as the mean ± SD (n ≥ 3). * *P* < 0.05, ** *P* < 0.01, *** *P* < 0.001


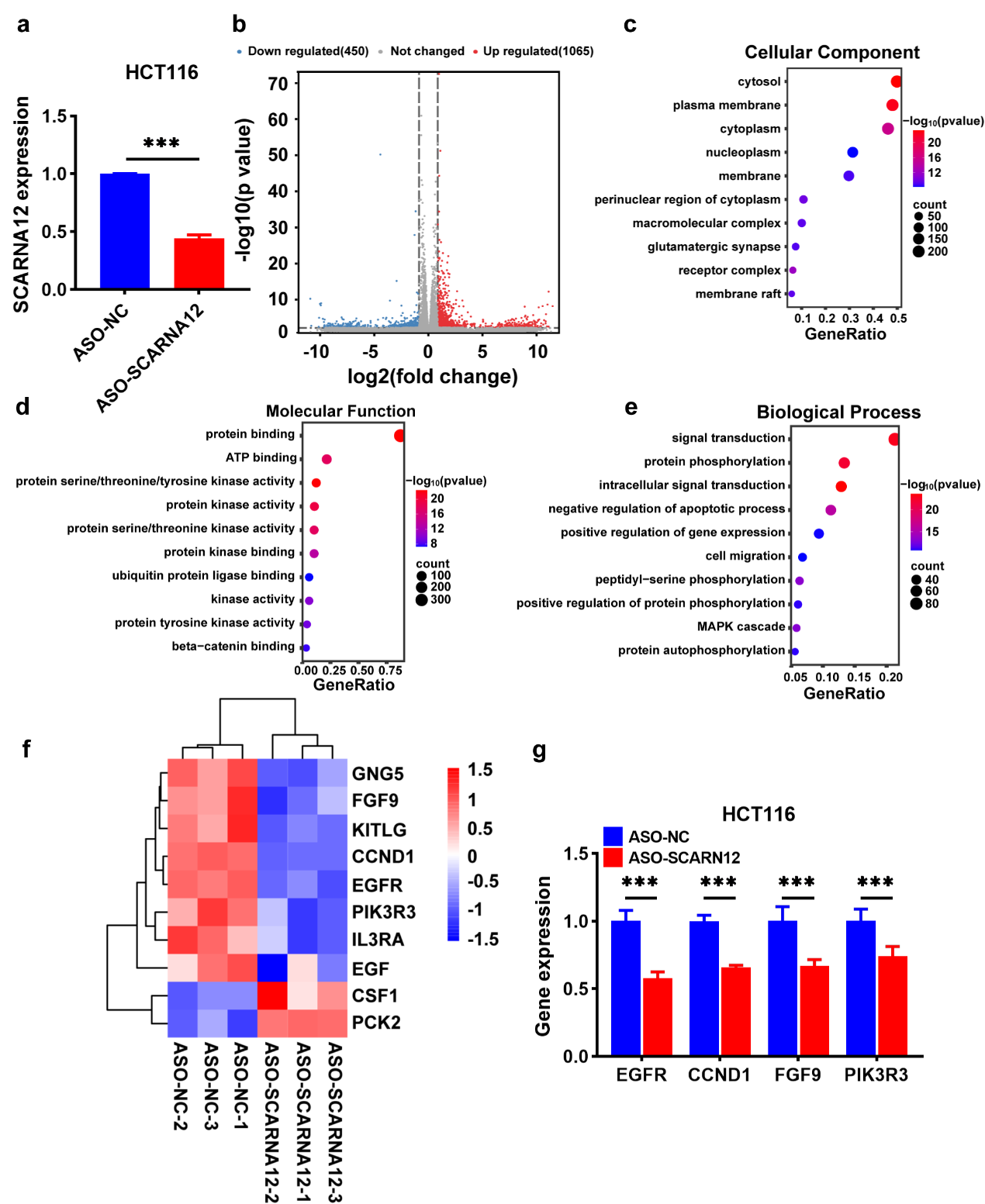


**Fig. S6** Exploring the underlying biological mechanisms involved in SCARNA12. **a** The knockdown efficiency of SCARNA12 in HCT116. **b** Volcano plot revealed the differentially expressed genes obtained from RNA-seq analysis of HCT116/ASO-NC and HCT116/ASO-SCARNA12 groups. **c-e** Top 10 strikingly enriched GO annotations for cellular component (**c**), molecular function (**d**), biological process(**e**). **f** The heat map presented representative differential genes determined by RNA-seq analysis in the PI3K/AKT signaling pathway. **g** The levels of indicated target genes verified by qRT-PCR. The results are presented as the mean ± SD (n = 3). ****P* < 0.001


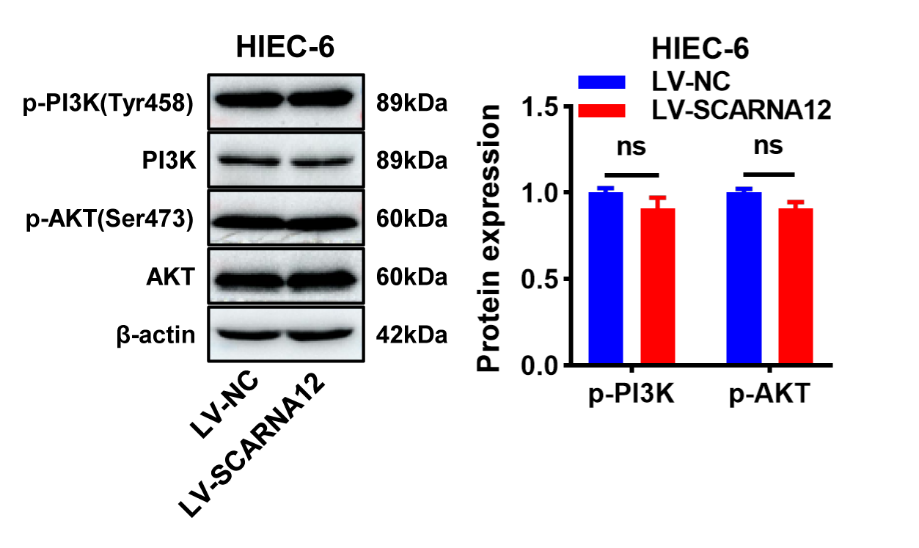


**Fig. S7** The proteins expression of PI3K/AKT pathway in HIEC-6. The results are presented as the mean ± SD (n = 3). ns, no significance

**Table S1** Primer sequences used in this study

| **Gene** | **Sequence** |
| --- | --- |
| SCARNA12-F | 5'-CATTTCTGGTGCTGCCCCTA-3' |
| SCARNA12-R | 5'-AGATCCAAGGTTGCGCTCAG-3' |
| U6-F | 5'-CTCGCTTCGGCAGCACA-3' |
| U6-R | 5'-AACGCTTCACGAATTTGCGT-3' |
| EGFR-F | 5'-CAGCGCTACCTTGTCATTCA-3' |
| EGFR-R | 5'-TGCACTCAGAGAGCTCAGGA-3' |
| CCND1-F | 5'-GCTGCGAAGTGGAAACCATC-3' |
| CCND1-R | 5'-CCTCCTTCTGCACACATTTGAA-3' |
| PIK3R3-F | 5'-ATGTACAATACGGTGTGGAGTATG-3' |
| PIK3R3-R | 5'-GCTGGAGGATCCATTTCAAT-3' |
| FGF9-F | 5'-CCAGGAAAGACCACAGCCGATTC-3' |
| FGF9-R | 5'-GTCCACTGTCCACACCACGAATG-3' |
| GAPDH-F | 5'-GGGAAACTGTGGCGTGAT-3' |
| GAPDH-R | 5'-GAGTGGGTGTCGCTGTTGA-3' |
